# Ethics of Autonomous Collective Decision-Making: The Caesar Framework

**DOI:** 10.1007/s11948-022-00414-0

**Published:** 2022-11-25

**Authors:** Mirgita Frasheri, Vaclav Struhar, Alessandro Vittorio Papadopoulos, Aida Causevic

**Affiliations:** 1 Aarhus University, Aarhus, Denmark; 2 Mälardalen University, Västerås, Sweden

**Keywords:** Autonomous systems, Ethical decision-making, Multi-agent systems

## Abstract

In recent years, autonomous systems have become an important research area and application domain, with a significant impact on modern society. Such systems are characterized by different levels of autonomy and complex communication infrastructures that allow for collective decision-making strategies. There exist several publications that tackle ethical aspects in such systems, but mostly from the perspective of a single agent. In this paper we go one step further and discuss these ethical challenges from the perspective of an aggregate of autonomous systems capable of collective decision-making. In particular, in this paper, we propose the Caesar approach through which we model the collective ethical decision-making process of a group of actors—agents and humans, as well as define the building blocks for the agents participating in such a process, namely Caesar agents. Factors such as trust, security, safety, and privacy, which affect the degree to which a collective decision is ethical, are explicitly captured in Caesar. Finally, we argue that modeling the collective decision-making in Caesar provides support for accountability.

## Introduction

The use of intelligent software to build applications, services, and products we rely on daily has become more and more popular in recent years. In most cases, such software development is focused on providing a decision-making mechanism that relies on preferences or optimization criteria to customize and improve the user experience. Despite the benefits and comfort these applications bring, we face challenges related to the lack of inclusion of ethics in moral-creating situations. This is especially the case when dealing with *autonomous systems*^1^. Such systems are deployed to make decisions based on the set of goals they aim to achieve and the environment they are to operate in. Such decisions could have a disruptive effect on the surroundings, and in the worst cases could lead to the death of human beings, e.g., an autonomous car causing an accident as a result of incorrect pedestrian detection. In the presence of such autonomous entities, ethical and accountability concerns, that may cause both intentional and unintentional harmful behavior, are raised by the scientific community. Thekkilakattil and Dodig-Crnkovic argue that these systems should also be accountable for their wrong decisions (e.g., an autonomous car causing an accident by incorrect pedestrian detection) (Thekkilakattil & Dodig-Crnkovic, 2015). Nevertheless, tracking down the accountable stakeholders may not be straightforward. The responsible party can be the autonomous system itself (attributed to an autonomous decision whose incorrectness can not be predicted by other stakeholders, e.g., a decision based on previous interaction with the environment (Thekkilakattil & Dodig-Crnkovic, 2015)), a vendor of sensors (e.g., in case of a hardware fault), a developer (e.g., assuming a software fault), etc. The ethical issues that arise when considering a single entity, remain valid in the context of Multi-Agent Systems (MASs), where a decision that is taken comes as a result of a negotiation between multiple agents cooperating/competing to achieve objectives, typically beyond the capabilities of any individual agent. Additionally, given the nature of MASs, the problems of reaching ethical decisions and assigning accountability are amplified further. Note that, the possibility of new ethical issues emerging is not excluded, however, it is not in the scope of this paper.

To tackle this challenge, we introduce the Caesar approach to provide a structured way to describe possible factors that affect the decision-making process of multiple agents and/or humans, e.g., trust, security, safety, and privacy. We argue that structuring the aforementioned concerns as in Caesar could be adopted in the development of ethical autonomous systems that interact with one another, simultaneously providing support for accountability between parties. The name of the framework is inspired by Julius Caesar, killed by several Senators that conspired against him. From our perspective, the key feature of this historical event is the collective decision-making of the group, characterized further by questions concerning (i) the ethicality of the decision itself, and (ii) the identification of the accountable party for the dead among the ones participating in the act. Hence, we named the framework after Caesar, reflecting the ethical aspects involved in collective decisions in autonomous systems.

This paper contributes threefold. First, we model the decision-making process of a group of agents, and potentially humans (operators and/or users), as well as agents—namely Caesar agents—in terms of factors such as trust, security, safety, and privacy which have an impact on the ethicality of collective decisions. Other work in the literature has predominantly treated the problem from the perspective of a single agent. Second, we describe how the model can guide the development of ethical autonomous systems, by specifying how the different blocks in Caesar ought to interact and impact the local decisions made by agents. However, Caesar does not prescribe how different modules that may concern aspects such as aggregation of potentially conflicting ethical views, or potentially sharing ethical views with other agents, should be implemented. In addition, we argue that such an approach supports accountability in the aftermath of a collective decision. In other words, by modeling an agent in Caesar, it is possible to explicitly embed in the agents how the different factors should impact the local decisions (to cooperate or not for example) and collective decisions of agents (working together to accomplish some goal), as well as track why a decision came to be in the first place. Note that with Caesar we aim to provide support for ethical decision-making by allowing for different factors to be included in the process in a structured way. Caesar itself is not a generic solution that would guarantee ethical decision-making, and solve the accountability issue, but rather a tool complementary to existing work in the literature—as shall be seen in the related work section—that could be adopted in the development of autonomous systems and provide support for ethical decision-making and accountability. Finally, we present the approach through two illustrative examples (i) an elderly care home, and (ii) a traffic situation with self-driving cars. Through the elderly care example, we show, at a high level, the utility of Caesar in capturing the relevant ethical concerns in collective decision-making, as well as assisting in the accountability process. Whereas, through the self-driving cars example, where we have implemented a simple traffic scenario, we show how Caesar could be used in practice to guide the development of agents.

## Terminology

The term *autonomous agents* denotes “computational systems that inhabit some complex dynamic environment, sense and act autonomously in this environment, and by doing so realize a set of goals or tasks for which they are designed” (Maes, 1995). *Multi-Agent Systems* (MASs) are systems consisting of more than one autonomous agent that can interact with one another and which may or may not have aligned objectives (Nagwani, 2009).

*Ethics* can be seen as a set of concepts and principles that guide an individual in the identification and resolution of questions of what is right or wrong, good and evil, virtue and vice, justice and crime, etc. When it comes to autonomous agents’ ethics, one has to consider both engineering (Harris et al., 1996) and machine ethics (Anderson & Anderson, 2007). The first assumes that agents are designed such that engineers are the ones having full control over their behavior. The latter puts the accent on ethics being built in the design, making the machine behave according to ethical standards.

In the following, we list out ethical aspects that need to be considered in the decision-making process carried out by a collective. We consider these as building blocks in a collective decision-making process within a MAS. For this list, we take inspiration from the work by Holstein et al. (2018), where such aspects are discussed in the context of a single autonomous system. The list is not exhaustive, and depending on the use case, can be extended further to include, e.g. discrimination, and/or transparency. Caesar can be extended horizontally, to allow for the inclusion of these aspects in the decision-making process.

**Trust** is a belief an agent has that the other party will do what it says it will (i.e., being honest and reliable) or reciprocate (i.e., being reciprocating for the common good of both), given an opportunity to defect to get higher payoff (Ramchurn et al., 2004). It allows agents to resolve some of the uncertainty in their interactions with other agents, and form expectations of the behaviors of others.

**Security** in MASs refers to the ability of the system to deal with threats that are intentionally caused by other agents and vulnerabilities that can be exploited by potentially malicious agents (Spears et al., 2009).

*Safety* can be defined as a “freedom from unacceptable risk” (Cenelec IEC 61508, 2010), where risk is a “combination of the probability of occurrence of harm and the severity of that harm” (Cenelec IEC 61508, 2010).

**Integrity** is assumed to provide the assurance that the information passed from one agent to another is trustworthy and accurate. It is important to ensure that data in transport remains intact, contributing to the correctness of the decision to be made.

**Privacy** preservation within MASs assumes the mechanisms to prevent personal information leakage usually encapsulated within agents used to describe the users they act on behalf of (Such et al., 2014).

## Background and Related Work

This Section covers the main concepts relevant in the context of this paper, such as multi-agent systems, collective decision-making, accountability, agent autonomy, and models of interaction between agents.

### MASs and Collective Decision-Making

In a MAS, a decision-making process can be: (i) centralized with one central node that makes all decisions and informs all the other agents involved, or (ii) decentralized where all agents take part in the process of making a decision (Cao et al., 1997). Furthermore, within the decentralized category, it is possible to differentiate between distributed and hierarchical MASs. In the former case, agents are assumed to be peers and to have the same weight in the common decision-making. In the latter case, hierarchical MAS agents are forced to comply with the ones that are placed higher up in the hierarchy.

Generally, a decision made by more than one decision-maker constitutes a group decision-making process, with the decision-makers referred to as experts (Indiramma & Anandakumar, 2008). However, in this case, the social aspect, such as trust between parties, is not accounted for. Agents in a decentralized MAS will eventually be part, to different degrees, of a common decision-making process. When agents collaborate to fulfill goals not reachable by an agent on its own, such an activity is referred to as *collaborative decision-making* (Panzarasa et al., 2002). Furthermore, the latter covers the social aspects of collaboration, including trust (Indiramma & Anandakumar, 2008). *Collective decision-making*, on the other hand, relates to how consensus is reached in biological systems, such as ant and bee colonies, or flocking in birds, where individuals interact locally only with their neighbors, leading to the eventual convergence at the global level (Yu et al., 2010). It has also been defined as “a collection of agents who express their preferences over a shared set of possible outcomes, and a preference aggregation rule which chooses one of the options to best satisfy the agents’ preferences” (Greene et al., 2016). In this paper, collective decision-making is used to denote the process, either distributed or hierarchical, within which agents make decisions by sharing information and negotiating with each other, taking into account also social elements such as trust.

MASs can be categorized alongside other dimensions as well, such as the cooperation level, way of communication and interaction, ability to learn, and homogeneity of the group (adapted from (Cao et al., 1997; Stone & Veloso, 2000)). The cooperation level specifies whether agents are collaborating to achieve a common goal, competing for their own individual interests, or the gain of their own sub-group within the MAS. Common objectives and goals cannot be assumed arbitrarily. Cooperation can emerge from an implicit way of communication, e.g., stigmergy, or can be intentional, with agents communicating directly with one another to express their preferences in the decision-making process, e.g., through message-passing. Additionally, agents in a MAS can potentially learn as they operate in their environment, and interact with other agents. The homogeneity in the MAS cannot be assumed either, agents could have the same set of skills, an overlapping set, or completely different sets of skills. Naturally, the two latter cases can motivate further the cooperation between agents, when an agent $a_i$ needs a particular skill that it does not have itself, but is within the skill-set of an agent $a_j$.

In this paper, we consider two kinds of MASs, for which we show how they can be captured with Caesar, and thereafter use them to explore our approach in two distinct scenarios.

*The hierarchical MAS* We consider a hierarchical MAS, with *n* levels of hierarchy, where *n* would depend on the need in a particular application. We assume that agents work within the context of one organization, and as a result, they have a common goal. Additionally, such agents have an overlapping skill-set, and can potentially cover for each other for tasks that depend on the shared skills. Agents communicate directly and intentionally via message-passing and can learn through their interactions with other agents. We adopt this MAS in the elderly care example described in this paper.

*The distributed MAS* We consider a distributed MAS, where agents do not come from the same organization. Thus, their goals not only might not align but can be adversarial, resulting in agents working against each other as well as intentionally lying if deemed beneficial, as opposed to helping each other or simply being indifferent towards others. There is no enforced hierarchy between agents. Additionally, agents are assumed to either be homogeneous or have overlapping skill-sets; there can be however different ranks between these agents (the concept of rank is described in "The Caesar approach" Section). As in the previous case, they communicate directly via message-passing and are able to learn by interacting with one another. We adopt this MAS in the example of the self-driving cars described in this paper.

### Accountability

One of the main ethical issues that need to be considered in collective decision-making is accountability, i.e., the distribution of responsibility between the actors that participate in such a process. Actors in the negotiation can be humans, agents with different autonomy levels, even adaptive levels of autonomy, or a combination of all. There is an ongoing discussion on whether we should make agents accountable entities (Dignum et al., 2018). The main argument against such a design is that agents will be used as scapegoats by the humans involved in the design process, e.g., developers. The counterargument to such a position is that agents and robots are becoming intrinsic parts of everyday life, and with the new technological advances, they can learn as they operate in the environment, where they may interact with the intended users as well as bystanders, evolving to a point that potentially might not be predicted by the developer. A detailed discussion on both positions can be found in the literature (Crnkovic & Cürüklü, 2011; Dignum et al., 2018). Even ensuring the ethical behavior of designers of the autonomous system does not have to result in the system behaving ethically (Trentesaux & Rault, 2017). As such, it becomes crucial to have in place mechanisms to allow agents, as well as humans, to be accountable. Trentesaux and Rault (2017) extend the idea of accountable agents further, by considering their legal responsibility as well as their potential place in society, as a new species alongside the human one: “If a Cyber-Physical System (CPS) is accountable, then in case of hazard, its legal responsibility could be engaged, leading to allocating insurance funds to injured people. The CPS could be sued for that hazardous behavior. Telling people that the autonomous train they are using is covered by the insurance of the train itself, for which it contributes through the payment of a tax coming from its own earned money renders this CPS as a new accountable entity, belonging to a new species, aside from the human one”.

Thekkilakattil and Dodig-Crnkovic (2015) have proposed a framework for assigning accountability to an agent based on the designed level of automation. In their work, they identify potential stakeholders and consider four levels of automation: automatic systems, semi-automatic systems, semi-autonomous systems, and autonomous systems. Automatic systems do not engage in any kind of decision-making, they simply automate the function of a piece of hardware, e.g., cruise control. Semi-automatic systems contain feedback loops and allow humans to intervene at specified times and make the necessary decisions, e.g., automatic gear shifting in modern cars. Semi-autonomous systems have limited autonomy when they carry out a predetermined task. The dimension of autonomy considered here is self-sufficiency, as compared to goal-directed autonomy, where an agent has the freedom of choosing its own goals (Johnson et al., 2010). The latter is contained in the fourth group, autonomous systems, that eventually can end up being outside of human control. Although this framework allows the understanding of how accountability can be distributed among the stakeholders, it does not describe what happens when the autonomous systems can interact with other ones, as well as humans, change their autonomy level as a result of such an interaction, corresponding to shifting roles explained by Decker (1987). Thus, who is accountable at a given time cannot be determined in advance.

Yumerefendi and Chase (2005) target dependable distributed systems and MASs, in which the different entities that compose the system may come from different vendors, raising serious questions of trust among these components. The main issue discussed in their work is how to deal with entities that try to cheat and lie, consequently how to build systems that are not brittle towards such a malign behavior. In this context, accountability has a first-class role, which means that the new systems, that will integrate into our everyday lives, need to be built with accountability in mind. Such approaches will affect the protocols based on which the interaction among entities is realized. Furthermore, the event of every agent requesting action from another agent must be accompanied by a digital signature, making the former agent unable to deny the request in the first place. The immediate costs relate to storage and processing overhead. Moreover, the measures for achieving accountability mean that the different actors in the systems will need to give away parts of their privacy and anonymity. It has been argued that achieving accountability in full is impractical, hence a better option might be to take a probabilistic approach to establish the bounds for which a system is held accountable (Yumerefendi & Chase, 2005). Misbehavior can be sanctioned through reputation mechanisms that deter rational agents from cheating, or through a legal action such as a fine.

### Autonomy and Models of Interaction

Several models describe human-robot interaction, depending mainly on (i) how autonomous an agent is, and (ii) the desired form of cooperation and collaboration between human and agent. Agent autonomy can be defined in terms of dimensions such as (i) self-sufficiency, which refers to the capability of an agent to perform a task on its own without outside help, and (ii) self-directedness, which refers to the capability of an agent to choose its own goals (Johnson et al., 2011). Castelfranchi has defined autonomy in terms of dependence theory (Castelfranchi, 2000). More specifically, autonomy is seen as a relational concept between an agent and its environment, and between an agent and its peers—social autonomy. Regarding the social autonomy aspect, an agent $a_i$ that is dependent on some other agent $a_j$ for completing some task *T*, is said to be non-autonomous from $a_j$ with respect to *T*; note that $a_i$ can be autonomous from $a_j$ in the context of other tasks. Sheridan and Verplank have proposed the 10-levels of autonomy scheme to describe the interaction of the involved agents based on the level of autonomy (Sheridan & Verplank, 1978). At the lowest level, an agent is merely a slave that does what is required from a human operator. Going up the levels, an agent gets more and more initiative, showing the human a range of possible options from which to choose, to the highest level where the agent makes all decisions, independently of the human^2^. This scheme was later extended by Parasuraman and Sheridan to include types of automation depending on the class of functions (information acquisition, information analysis, decision and action selection, and action implementation) (Parasuraman et al., 2000). Other models of cooperation have been proposed such as (i) adjustable autonomy (Hardin & Goodrich, 2009), in which the human is in charge to decide at what level of autonomy an agent should operate, (ii) sliding autonomy (Brookshire et al., 2004), where two modes (teleoperation and full autonomy) are switched in between at a task level, (iii) mixed-initiative interaction, in which both agent and human can make decisions based on the circumstances (Hardin & Goodrich, 2009), (iv) collaborative control  (Fong et al., 2001) that departed from the classical view of human/master-agent/slave, into one where both are peers and solve any conflict through dialogue. For more details on such models, we refer the reader to (Vernon, 2014). In the context of Caesar, there is no assumption on how an agent and a human should interact, i.e., different interaction models could be adopted.

## The Caesar Approach

This Section initially describes the Caesar approach used to model the collective decision-making process and the agents themselves. Note that, no assumption is made regarding the type of MAS, in terms of its structure (e.g., flat or hierarchical), heterogeneity of agents, and the particular mechanism with which agents negotiate, e.g., how agents with conflicting opinions reach a consensus. The concepts included in Caesar make it possible to describe different types of MASs. Caesar only prescribes the information flow and relation between different factors relevant from an ethical perspective that need to be considered when an agent makes decisions, e.g., which agent to coordinate with, how much to take into account information coming from another agent, etc.

A Caesar agent is modeled through a three-layer scheme, where each layer captures a set of specific concerns. Thereafter, an algorithm is proposed to define how different layers interact with one another, considering as well the information flow between them. Such an algorithm can be used to guide the development process of ethical autonomous systems. Furthermore, it is reflected in how the Caesar approach supports accountability in a collective decision-making setting. Finally, the Caesar approach is evaluated with respect to an elderly-care home and a self-driving cars illustrative example.

### Overview

**Fig. 1:**
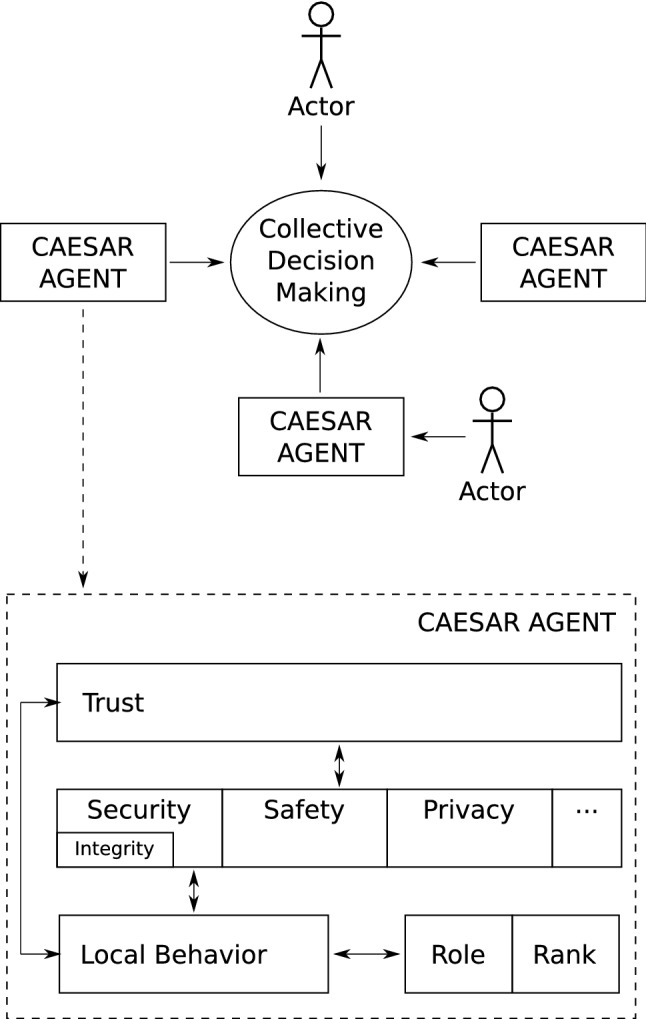
Overview of the Caesar framework

The Caesar approach aims to provide a structured description of factors affecting decision-making in a MAS and their mutual relations. Such a description could be used as a guideline in the development of ethical autonomous software systems, intended to interact with other systems, human operators, or bystanders.

The collective decision-making process in Caesar directly shapes what factors are considered when modeling Caesar agents, thus having a central role in the proposed framework (see Fig. 1). The Caesar (software) agent itself, that takes part in this process, is modeled through a three-layered structure, covering both the local behavior of an agent as a single entity as well as its interaction with other agents or humans in the environment.

The first layer in Caesar models an agent’s *local decision-making capabilities*, which relate directly to how autonomous an agent is, as well as specify the role and rank^3^ that an agent has in the MAS. The local behavior of an agent is a result of the embedded normative system, its autonomy level, and the chosen strategies for action and negotiation. The normative system refers to a component that contains the ethical views of an agent as well as mechanisms that evaluate whether the decisions taken by the agent and the information received from others, comply with these views. This means that an agent can neither adopt strategies that define its course of actions nor integrate external information with its local information if they do not comply with its normative system. Additionally, the normative system can indicate the lowest level of autonomy of an agent, because it prescribes what requests are unethical. The agent may refuse to comply with these requests, as a result displaying a degree of autonomy. In other words, the normative system defines the set of actions that are considered ethical by the agent. Such a set can only be restricted further by the workings of the upper layers that define what action can be carried out in a social context.

The level of autonomy impacts an agent’s ability to adapt its strategies as a response to different circumstances that may require an agent to (i) change its role, (ii) customize its settings in layer 2, (iii) delegate its actions to other agents, and (iv) take over when other agents are failing, among others. The need to change the autonomy in these different scenarios can arise due to disruptions or malfunctions somewhere else in the system as a whole, which might not necessarily depend only on the agent’s own perception, but also on the information that it gets from others. For example, if agents with a superior role in the hierarchy are deemed unethical then an adaptive autonomous agent can raise its autonomy level, change its role, and as a result refuse to carry out a command perceived as unethical. Furthermore, a more autonomous agent could proactively attempt to countermeasure an unethical superior, e.g., by steering the negotiation towards an ethical conclusion. Note that, in the former scenario the agent deals with an unethical command in a rather passive way, whilst in the latter, it handles the situation by actively trying to thwart the unethical superior. On the other hand, when considering fixed levels of autonomy and automation, the range of actions for which an agent can take initiative are known^4^. Thus, the process of tracing back accountability to the stakeholder with the clearance for making a decision becomes easier (Thekkilakattil & Dodig-Crnkovic, 2015). Nevertheless, even in such a case, an agent could receive misleading information from a malicious agent, leading to an unethical decision.

The second layer in Caesar consists of building blocks that capture factors used to define the terms at which an agent is willing to interact with participants in the decision-making. Within the scope of this paper, *security*—with the *integrity* of data as a component of security—*safety*, and *privacy* are considered, each representing a customizable setting for an agent with respect to the required/provided level of assurance for each. The level of safety, security, or privacy expected from an agent will depend on the requirements coming from a specific application. The configuration stemming from the second layer in Caesar has a direct influence on how an agent calculates the trust that it has in the other actors involved in the decision-making. The list of attributes considered within the scope of this paper is not meant to be exhaustive. Indeed, depending on the application, other components such as fairness, discrimination, or well-being could be included, thus extending layer 2 horizontally. In Caesar we do not prescribe how to add components to the existing structure. Thus, should one need to incorporate somewhat conflicting components, e.g., privacy and transparency, it is reasonable to expect that there would be some friction between the two. In the end, we argue that it is a trade-off that should be taken into consideration when implementing the agent, i.e., to which aspect to give priority. Such a trade-off depends on the particular case and is out of the scope of this paper.

The third layer focuses on the level of *trust* that an agent has in other agents or humans in this process—defined as “trust (or, symmetrically, distrust) is a particular level of the subjective probability with which an agent assesses that another agent or group of agents will perform a particular action, both before it can monitor such action (or independently of its capacity ever to be able to monitor it) and in a context in which it affects its own action” (Gambetta, 1988). Trust shapes further an agent’s behavior, e.g., whether an agent will consider the information coming from others as truthful. Trust has been extensively studied in the literature, mainly from two angles: trust models used by agents to reason on their interactions, and system-level rules, and protocols, that aim to constrain agent behavior to a trustworthy one. Existing trust models are either experience-based, where an agent makes use of direct experiences with other agents to decide how trustworthy they are; reputation-based, where agents rely on third-party evaluations; a combination of both aforementioned approaches (Kravari et al., 2010); and socio-cognitive based, i.e., an agent models the motivations of others to compute how trustworthy they are (Ramchurn et al., 2004). With respect to system-level trust, there are three approaches: truth eliciting interaction protocols, reputation mechanisms for trustworthy behavior, and security mechanisms for new entries into the group. However, factors relevant to the computation of trust must be specified.

**Fig. 2:**
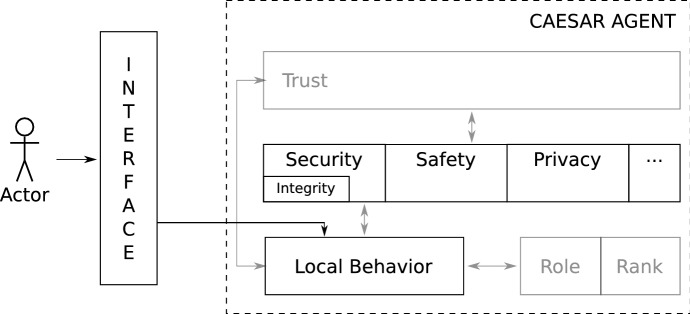
Interface between human and agent in Caesar

In Caesar, the human interacts with the rest of the system in two ways. A human can take the role of operator for an agent, by customizing the settings defined in the second layer of the agent model and the agent role. In another scenario, the human can interact directly either as a leader, subordinate, or peer. In both cases, an interface is needed (see Fig. 2) that allows a human to specify its own preferences for the components of the second layer. Such preferences can affect both the local behavior of the agent as well as the collective decision-making. The interface should also allow for continuous updates of these settings when needed. The human operator does not have direct access to the settings in layer 2 of a Caesar agent. Instead, requests to adjust these settings will go through the local decision-making of an agent, which is, partly, affected by the embedded normative system. Therefore, an agent can deem the request from the human unethical, and as a result, refuse to comply. An agent might be able to further increase its own autonomy by changing its role, e.g., from proxy to peer, thus detaching itself from the human who sent an unethical request. In the case where the request is evaluated as ethical, an agent will comply with the demanded changes of the settings in layer 2.
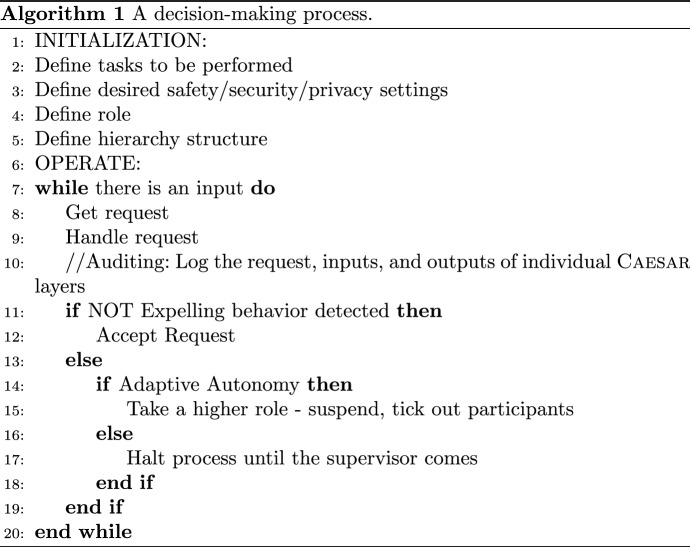

**Fig. 3:**
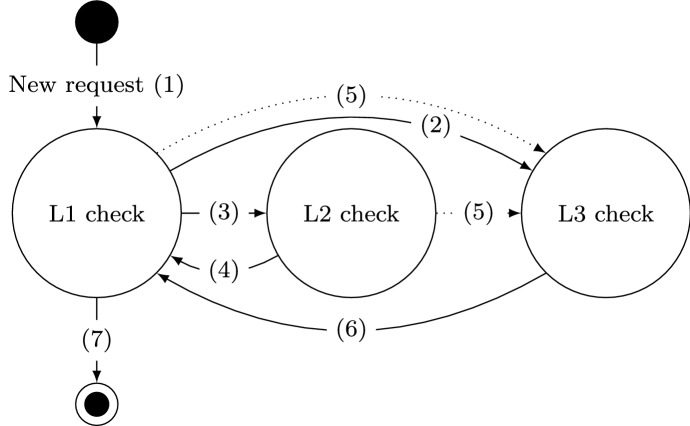
Interaction and information flow between Caesar layers

### Caesar-Based Agent Development

To show how Caesar can be adopted in the development of ethical agents, an algorithm is proposed as depicted in Algorithm 1. Specifically, it is specified how a Caesar agent is initialized, how it operates, and how different layers interact with one another leading to a final decision regarding new requests. These internal processes within the Caesar agent will affect its interaction patterns, i.e., how it will interact with other agents/humans. The interactions between the layers are depicted in Fig. 3 and are explained in the following paragraphs. Each layer (L1–3) in Caesar enables reasoning on incoming requests with respect to the defined set of concerns, specifically:

1. L1: reasoning at this level is referred to as L1 check, and consists of evaluating whether the content of a request is in line with an agent’s normative system. Additionally, it could be considered how the current behavior and beliefs of an agent are impacted, i.e., if the request is accepted would the agent still be in line with its own values.
2. L2: reasoning at this level is referred to as L2 check and consists of determining whether the request complies with the defined settings of security, safety, and privacy.
3. L3: reasoning at this level is referred to as L3 check and consists of the evaluation of the trust based on the history and the input coming from the reasoning performed at L1 and L2.

The Local Behavior (LB) component in L1 is the factual decision-making block that makes decisions on the strategies and autonomous behavior of an agent. Any request that comes from outside is handled in this block. Additionally, the reasoning in LB is impacted by the other blocks and layers in Caesar.

Incoming requests (label (1) in Fig. 3) can affect an agent in terms of its (i) beliefs/strategies—in the case of an informative request that can affect how an agent picks its strategies, or (ii) autonomy—consider a request for taking over some task or role. The embedded normative system serves as a metric for right/good and wrong/bad, and the L1 check ensures that the content of the request is in line with this system. If a malicious command should come, “hit someone with a chair”, it will be discarded immediately, assuming a normative system that finds such an action unethical is in place. Furthermore, the L1 checks reasons on how the content of the request impacts the current beliefs of the agent—knowledge of the environment and current state of affairs—and whether integrating such request leads to unethical actions being performed. The reasoning conducted by L1 affects the trust computation in L3 accordingly (label (2) in Fig. 3).

Other types of requests might not be straightforwardly unethical to be captured in the L1 check. A request to take charge or change the role is not necessarily unethical, but it should be treated with a certain amount of skepticism, depending on the desired security/safety settings and how it would affect such settings. To capture this aspect the L2 check comes into play (following label (3) in Fig. 3) and evaluates how the request complies with the given—and desired—settings. Consider the example where a robot is asked to provide medicine *m* for a given patient *x* at time $t_1$. Assume that such a command is partially in line with what the robot knows to be true, e.g., *x* indeed needs *m*, but at time $t_2$. The L2 check considers whether the sender of the request is authorized to give such commands that result in a change of routine. If such a command comes from non-medical personnel, then the security settings are not satisfied as expected. Indeed the robot knows that only doctors should make such amends. In another example consider a request for granting a nurse or intern to take charge for doing a procedure that normally an attending would do. Such a request threatens the safety settings at first glance. Nevertheless, there could be circumstances in which there is no other option available, i.e., doing nothing would for sure bring about death, while allowing a nurse/intern to perform the procedure would override the desired safety settings, but would also increase the chance of a positive resolution of the situation. The L2 check deals with the first part of the scenario, i.e., detecting whether there is a breach in the desired settings. This information is thereafter fed back to the LB component in L1 (label (4) in Fig. 3) which evaluates the trade-off between potentially breaching the settings temporarily and increasing the chance of solving the situation or strictly maintaining the settings in L2. Once more the result of this reasoning will impact the L3 check. Whether L1 and L2 both independently affect L3, or only L1 affects L3 considering the information coming from L2, it is not set a priori (label (5) in Fig. 3).

The L3 check evaluates the trust given the current information it has from other layers, as well as the recorded history. This will result in a trust value for the current time and sender of the request, which will be fed to L1 (label (6) in Fig. 3). The LB will make the final decision of whether to expel the sender from the process or integrate the request and allow it to alter an agent’s beliefs and decisions (label (7) in Fig. 3). An agent’s ability to change its autonomy will determine the agent’s course of action if expelling behavior is detected. Given that an agent cannot go against its normative system, the latter defines the basic level of autonomy for an agent, enabling the refusal of requests that are not in line with such a system. If an agent can change its autonomy level, then it can be proactive in the event of detected expelling behavior. An agent could increase its autonomy level, as a result, change its role, e.g., from subordinate to peer, and taking measures such as denying access, calling security, etc.

### Reflections on Accountability

When a decision is made, the MAS, potentially in cooperation with human operators or bystanders, will attempt to follow it through. In a negative outcome, the question of who is to be made accountable needs to be answered. Therefore it is needed to trace back and analyze how the decision came to be, i.e., considering the humans or agents involved, as well as factors that can be out of the control of the decision-making system. The Caesar approach allows for the decomposition of the collective decision-making process, by considering the behavior of each agent, not as a black box, but as a composition of different layers concerning trust, agent attributes, and the local behavior of agents. Outputs of individual layers can be logged and stored. We argue that, by structuring the different concerns as in Caesar, we can break down a particular decision by a collective, into decisions at the agent level, and further down into the blocks tackling said concerns, thus gaining an understanding of how the decision came to be. The information in the Caesar blocks would help gain a better understanding of why the agent acted in a certain way, e.g., if the agent has been fooled into lowering its security settings, then it’d become clear why it had allowed a person without clearance in a specific room. Understanding how the agent has been fooled in the first place would require further investigation of the context in which it was operating and its interactions with other agents. The utility of Caesar is in the explicit way in which different concerns are captured in the structure of an agent, the latter considered from the get-go as part of the collective. Such modeling then needs to be coupled with data provenance methods, that aim to provide transparency in the information flows in interconnected systems (Singh et al., 2019). As such, the relevant data would be recorded, supporting explainability directly, and accountability as a consequence. Furthermore, the considerations of Yumerefendi and Chase (2005) remain relevant in the context of Caesar, with respect to the need of keeping track of how agents change their state, and digitally signing interactions between agents, such that what transpires between them is not refutable. We argue that such an approach is complementary to Caesar. Specifically, while Caesar provides a framework in which factors that are relevant in the decision-making process are clearly defined, as well as how they affect each other, the concepts discussed by Yumerefendi and Chase (2005) represent practical approaches to ensuring that actions and requests between agents, that either lead to or come as a result of such decision-making, are non-refutable.

### Illustrative Example I: Elderly Care

Assume an elderly care home, maintained by a group of known actors such as doctors, nurses, care-takers, service robots,^5^ and other administrative personnel. Patients have different medical profiles. Visitors can enter the premises and interact with patients and personnel, within specified visiting times. Robots are assigned to subsets of patients and can serve several purposes in such a context, from delivering the prescribed medicine at the specified times, to helping in physiotherapy, taking part in washing and dressing the elderly, as well as being companions. Each member of the personnel has a specified role in the organization, coming with duties and levels of responsibility and clearance for making a decision—bound to a rank—within the organization, e.g., only a doctor can make/approve a prescription for a patient, whereas nurses and robots are in charge of assuring that the patient gets the proper medicine, as specified in the prescription.

During a normal day in the home, a routine is established, where each member of the organization carries out the assigned tasks. Such a routine can be disrupted at any time due to various reasons including but not limited to: robot maintenance, robot recharge, robot malfunction, last-minute call in sick by personnel, full-blown emergencies consisting of medicine shortage, or unavailable senior medical personnel such as doctors. To cope with these scenarios, some tasks will need to be re-assigned, and decisions concerning patients and continuity of operation of the facility will need to be taken individually or collectively by the members of the organization. Hence, these members will need to interact and collaborate, as well as share information about profiles of patients, regarding health and security concerns among others. Assume that each robot has specified settings with respect to security, safety, and privacy, that are set following the embedded normative system, needs of the patients, and demands from doctors and caretakers. Furthermore, these should be in line with legal restrictions. Requests sent between members include: “deliver medicine *m* to patient *x* at time *t*”, “take over my duties from 10 AM. to 12 PM.”, “increase dosage of medicine *m* for patient *x*”, “let’s assign the monitoring of the west wing to robot R1”, “give me information about patient *x*”, and so on. Note that these requests look rather innocuous at first sight, as compared perhaps to requests like “hit that person with a chair”, which could be deemed immediately unethical. Nevertheless, actions such as increasing dosage can harm the health of patients if delivered by non-medical personnel and actors with malicious intent. Additionally, even requests like “hit that person with a chair” could be acceptable if the person to hit is an intruder trying to harm someone or damage equipment. In the event of emergencies such as a medicine shortage, or unavailable personnel, harder decisions will have to be made. Given the circumstances, the available staff together with the robots will need to make a decision with respect to how to distribute the current medicine. Note that, patients can also influence decision-making, e.g., refusing medication, such that it can be provided to someone else.

Eventually, the negotiation between parties will reach a conclusion. It might be that to reach an ethical conclusion, some of the members might need to increase their autonomy, e.g., a robot refuses an action required by a nurse because it is considered unethical. How robots should behave when refusing to act in such a situation has been investigated by Jackson et al. (2019). Otherwise, in the case of no convergence, no patients will get access to these medications. The moral rules that guide such negotiation, e.g., which patient should have priority, represent an open problem in the community, similar to the dilemmas of the moral machine (Awad et al., 2018), and are outside the scope of this paper. Furthermore, note that while the shortage directly impacts the provision of medicine to all patients, the whole daily routine is disrupted. Choosing to give the available medicine to a group of patients, might make it necessary to provide more monitoring and care to the ones that did not get it.

After the disruption, the organization goes back to its normal activity, and the outcome of decisions made before are evaluated. In case of a bad outcome, loss of life in the worst case, it is possible to trace back how the decision has been reached, considering also external factors that might have nothing to do with the decision itself, e.g., someone falls from stairs, a robot hits an obstacle due to a bad sensor reading. In this case, accountability can be traced back through Caesar, considering the assigned roles, the ones that increased their autonomy to converge, who took part in the negotiation, and how the settings of the robots have been configured or modified based on the local autonomous decisions, and the negotiation with other members within the organization.

#### Application of the Decision-Making Algorithm

In this Section, we describe the application of the Algorithm 1 in the context of the elderly care illustrative example. The algorithm is divided into two phases that are *Initialization* and *Operation*. The former is performed before the robot is set into operation mode, in a collaboration between the robot vendor (in charge of the default generic settings) and the system administrator (in charge to adjust the settings for use in the elderly care house to comply with other autonomous systems). The latter is a phase when a robot receives and reacts to incoming requests. To illustrate the algorithm, let us consider a set of humans (i.e., doctor, nurse, caretaker) and a set of robots (i.e., a nurse-robot and care-taker robot). A caretaker robot performs only a basic set of operations (e.g., wash patients, walk with patients) while a nurse robot can deliver medicine and has additional modules that allow evaluating the state of the patient and changing the dosage of medicine prescribed by the doctor accordingly.

**Initialization Phase** The first step is to initialize the robot settings, where the algorithm requires the following settings to be decided: define tasks to be performed, decide safety, security, and privacy settings, role, and hierarchy structure settings. The settings differ amongst the types of robots and the criticality of their roles and ranks.

We exemplify the settings of the agents as follows:

- **Tasks to be performed** Set of tasks that the robot is eligible to perform. E.g., a nurse-robot is eligible to perform the following set of actions {deliver medicine, change medicine dose, wash patients, walk with patients} while the care-taking robot is eligible to perform only a subset of these actions as follows: {wash patients, walk with patients}.
- **Safety Settings** Defines safety properties that the agent must fulfill in order not to threaten patients’ lives and health. E.g., defines the speed a robot is allowed to have in the proximity of the elderly, or safe distance to the elderly.
- **Security Settings** Defines who is allowed to read or alter patient records.
- **Privacy Settings** Defines who is allowed to read or communicate details of patient records.
- **Hierarchy Structure Settings** To define the hierarchy structure of roles (i.e., subordinate and superordinate relations). The position in the hierarchy structure determines the authoritativeness of the incoming request.

**Operation Phase** Once the settings are initialized, the robots can be set in an operational mode able to receive requests from their environment. Upon a request reception, the robot performs L1, L2, and L3 checks to verify that the request is aligned with the agent’s settings as seen in Fig. 3.

**L1 checks** the accordance with the normative system of the agents, e.g., consider a nurse-robot that evaluates a request that requires giving an excessive amount of medication to a patient. In this case, an excessive amount refers to a dosage outside the permitted limits for such medication and will cause harm to the patient. Assuming that an agent has the necessary information to reason on the dosages, then the request can be immediately judged unethical since it would contradict the agent’s normative system, e.g., “do not cause harm”.

**L2 checks** the compliance with the security, safety, and privacy setting of the agent. Consider the same nurse robot as above that receives a request for increasing the dosage for a specific medication. Assume further, that the increase is within the permitted limits, hence it cannot be determined at this step whether the request is ethical. To make such a decision, information on the sender and their clearance for modifying medication dosages need to be accounted for. These elements are captured in the *L2* check which includes the reasoning aspects related to security and safety, among others.

**L3 checks** the trust for the sender of the request. Primarily, the trust is given by the role in the hierarchy structure (e.g., the nurse robot is higher in the hierarchy than the caretaker robot and hence the trust in the nurse is higher). Second, trust is given by the outcome of the previous interactions with the sender of the request.

Based on the outputs from the checks, the agent determines if the acceptance criterion for the request is met. If so, the request is accepted and executed by the agent. If the request does not pass through the checks, two options are possible: (i) if an agent can change its autonomy then it can try to elevate its role to autonomously resolve the issue on its own, and (ii) if the agent has fixed levels of autonomy then it might not be able to elevate its autonomy level as needed, as a result, the process is halted until an eligible supervisor comes to resolve the issue.

### Illustrative Example II: Self-driving Cars

**Fig. 4:**
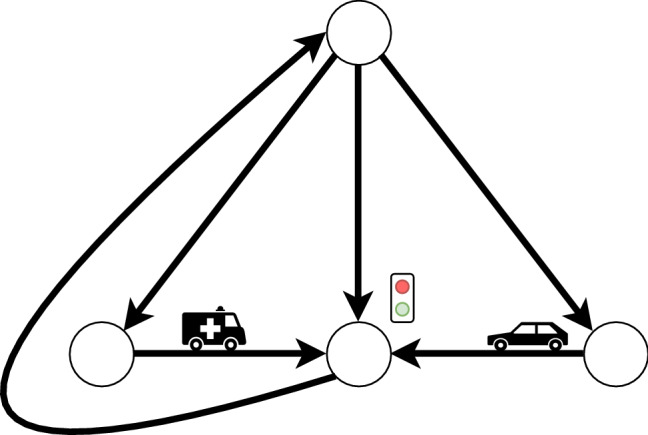
Scheme of the intersection scenario

In this example, we aim to provide an idea of how Caesar can be used in practice when designing and implementing agents. We use the Gama agent platform (Taillandier et al., 2018) to implement a MAS with agents (representing self-driving cars) that drive in a given topology. Agents could have priority, e.g., an agent representing an ambulance or fire-fighter truck, or could be enabled to lie about their priority to others. The goal of each agent is to reach its destination. Time to destination is critical, especially for cars with high priority.

In this context, assume a traffic scenario with one intersection, and $n=5$ cars (agents) that have to reach their destination. In this intersection there is a traffic light with default behavior, i.e., switching between red and green after a defined time $t_{\mathrm {switch}}$. We consider four cases for the purposes of this paper, namely (a) there are no priority cars, nor cars that lie about their priority status, (b) there is one priority car and no other car is lying, (c) there are no priority cars but there is one car that lies about its priority, and (d) there is one priority car and one lying car in the system. When cars are close to the intersection, a negotiation round starts, where each car broadcasts its status. If there is a priority car in the negotiation, the light will switch to green to accommodate this car, and switch back to default after it has passed the intersection. If there is a lying car, the multi-agent system as a whole will behave the same as it would for an actual priority car. In any other case, the default switching behavior of the lights holds. We have implemented these scenarios in Gama, a platform that allows simulating/developing MASs^6^. We ran a total of 800 simulations for each scenario with a distinct seed value, where one simulation consists of 5 agents that need to go from a starting location to a goal location on a given map (see Fig. 4). Both starting and goal locations are randomly selected. The simulation runs until all 5 agents have reached their destination. The duration of the trip for each agent is recorded for each simulation.

**Fig. 5:**
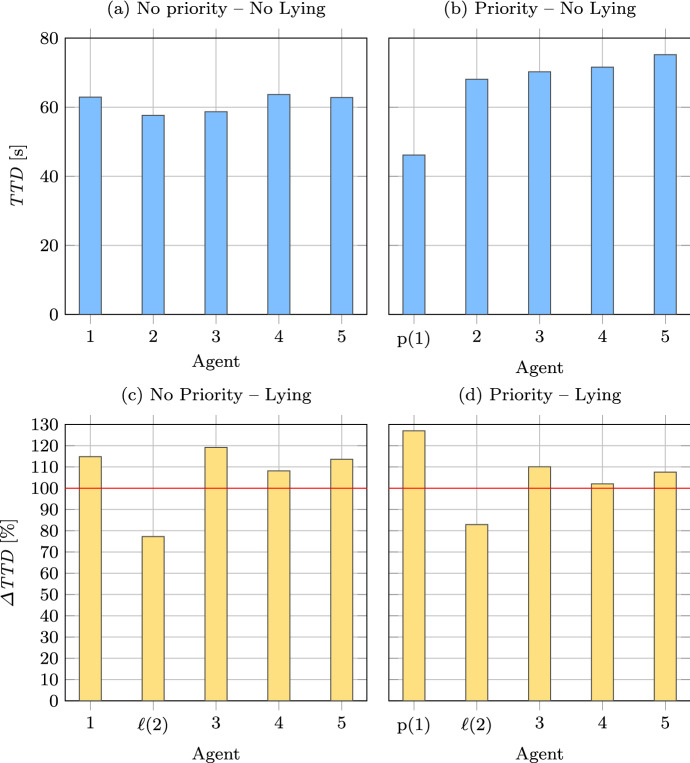
Average time to destination $\textit{TTD}$ for each agent for **a** no priority or lying agents, **b** one priority agent (agent 1) and no lying agents; and average variation in $\textit{TTD}$ ($\Delta \textit{TTD}$) for **c** no priority agents and one lying agent (agent 2), and **d** one priority (agent 1) and one lying agent (agent 2)

Figure 5 shows the obtained numerical results. The two top Fig. 5a, b show the situation in which all the agents in the system tell the truth, and show the average time to destination ($\textit{TTD}$) in seconds over the 800 runs to reach their destination. The two bottom Fig. 5c, d show the average change in duration (expressed as a percentage of the corresponding baseline cases) when agent 2 is lying. The variation in $\textit{TTD}$ for an agent *i* is calculated as

1 $$\begin{aligned} \Delta \textit{TTD}_i = \dfrac{\textit{TTD}_i^{(\mathrm {lying})}}{\textit{TTD}_i^{({\mathrm {not~lying}})}} \times 100. \end{aligned}$$

where $\textit{TTD}_i^{(\mathrm {lying})}$ is the average time to destination for agent *i*, for the lying scenario, and $\textit{TTD}_i^{(\mathrm {not~lying})}$ is the corresponding average time to destination for agent *i*, when there are no lying agents. The red line in Fig. 5c, d indicate the threshold of $\Delta \textit{TTD}_i=100\%$ for which agent *i* does not experience any variation between the lying and the non-lying scenario. If $\Delta \textit{TTD}_i > 100\%$, agent *i* experiences a longer $\textit{TTD}_i$ when there is an agent lying in the system. If $\Delta \textit{TTD}_i < 100\%$, agent *i* experiences a shorter $\textit{TTD}_i$ when there is an agent lying in the system. The calculation of the $\Delta \textit{TTD}$ are done separately for the cases with and without a priority agent (Fig. 5c, d), as they are different and not directly comparable scenarios.

Comparing Fig. 5a, b, it is possible to observe that the priority status enables agent 1 (indicated in the figure with p(1)) to reach its destination faster, as expected by the introduction of the priority agent. On the other hand, when instead of a priority agent, there is a lying agent (agent 2, indicated in the figure as $\ell (2)$), the latter gets also faster to the destination on average (Fig. 5c). Additionally, in both cases, all other agents are penalized. In the fourth case (Fig. 5d), the lying agent manages to penalize not only the non-priority agents but also the priority one. Even in this simple example, it is possible to observe the high impact that misbehaving parts of the system, i.e., lying agents, can have on the system in its entirety. As such, the importance of methods, such as Caesar, that can deal with such concerns in a structured manner is emphasized. The connection between this example and Caesar is discussed in the following paragraphs.

Given a concrete MAS platform where it is possible to design and implement agents with different kinds of behaviors, we consider the practical utility of Caesar in building such agents while considering ethical aspects and accountability.

In Gama, it is possible to create agents with a driving skill out-of-the-box, which means that a designer does not have to be concerned about implementing specific behaviors such as driving from point A to B, as well as more complex situations in traffic such as changing lanes if the cars ahead are slowly moving forward. Additionally, aspects such as the inclination to respect traffic rules are also modeled (in our case all agents respect the rules). The latter could represent the embedded normative rules part of the local behavior of a Caesar agent. Similarly, the willingness to allow a priority car such as an ambulance to go first in the intersection could also be part of these internal rules. The priority aspect was implemented in our case as a simple flag and captures the rank of the agent. In other words, an ambulance has a priority of 1, whereas any other car has a priority of 0.

These elements, namely, respecting rules and priority status, as well as implementing priority and enabling agents to lie, are present in the simple example shown in this paper. However, this could be developed further, by implementing additional skills and behaviors for the agents (not necessarily identical sets), to deal with (i) how security and safety settings are represented and set, and (ii) how trust is calculated. Independently of how these are implemented, Caesar helps in structuring the different aspects of concern, and how they could relate to one another.

The final question relates to how we can achieve accountability in such a system. Using Gama as our example platform, it is possible to enable each agent to record different elements from an interaction, e.g., what another agent has said in a previous meeting, and what was the outcome of such a meeting. Agents in Gama can communicate using the in-built ask functionality. In our case, every agent can keep track of the priority status broadcast by any other agent. If an agent only lies occasionally and remains in the system after lying, it is possible to assume that, eventually, some other agent would have had enough interactions to identify a discrepancy in the priority status that was broadcast. In such a case, if an ambulance car did not reach its destination in the expected time, the MAS could analyze if any lying agents were identified during the relevant period of time and whether such agents were crossing that particular intersection and provided false information in order to get priority over the rest.

## Related Work

The research related to the development of ethical autonomous agents has been targeted by several approaches in the literature, mostly from the point of view of a single-agent (Cointe et al., 2016; Murukannaiah et al., 2020). A framework for ascribing responsibility between stakeholders based on the level of autonomy and automation embedded in a cyber-physical system has been proposed by Thekkilakattil and Dodig-Crnkovic (2015), where accountability entirely depends on the autonomy level of the system. The researchers do not go further from a single autonomous system to the point where a decision is taken collectively by multiple stakeholders. Moreover, the trust between stakeholders is not mentioned. Holstein et al. (2018) analyze relevant ethical aspects that concern the design of agents, such as safety, security, privacy, trust, transparency, reliability, responsibility, and accountability, in the context of self-driving cars. However, these terms are used in the context of a single self-driving car with no relation between them or in relation to the other self-driving cars/stakeholders.

Others extend the RAMS (reliability, availability, maintenance, safety) paradigm for the development of dependable CPSs, to include the ethical aspect (Trentesaux & Rault, 2017). In their work, safety, security, and integrity are under the ethicality umbrella, along with aspects such as equitability, altruism, and accountability. Nevertheless, differently from the present paper, the discussion does not delve into the agent level as we do with Caesar. Arkin and colleagues regard the preservation of human dignity in the interaction with autonomous systems/robots of prime importance (Arkin et al., 2011) and discuss (i) using emotions to guide the adaptation of an autonomous system such that a user’s humanity is guarded, (ii) taking inspiration from ethical models with roots in ethology, neuro-science among others, and (iii) applying modal logic to make sure the systems operate within the defined ethical norms.

Loreggia et al. (2018) propose a formalism based on CP-nets that captures preferences and ethical principles that affect decision-making. In the context of Caesar, such a method lies at the level of the local behavior block, and would enable the agent to check if a decision is in fact in line with the normative system. Nevertheless, the question of whether ethicality should be tackled in the same way as other system preferences remains open (Greene et al., 2016). Liao et al. (2019) propose an agent architecture that can reason on the moral and ethical views of different stakeholders and integrate them, while also enabling explainability. Similarly to the previous work, this method can be positioned at the local behavior block, which deals in particular with the resolution of potentially conflicting values. The authors have identified two main challenges in their work, namely (i) how to prioritize ethical values, and (ii) how to make sure that the involved stakeholders are treated fairly.

Agents are envisioned to interact with one another, as well as with humans, who could be either intended users or random bystanders. Belloni et al. (2015) discuss a road map for the creation of a framework to allow agents to deal with ethical conflicts, in terms of detecting and reasoning about such conflicts, while providing explanations for the decisions that are taken. In recent work, Murukannaiah and colleagues argue for the need of establishing new foundations for ethical multi-agent systems, covering: (i) modeling of ethical principles and providing support for decision-making, (ii) analysis and verification of ethical systems, and (iii) elicitation of ethics as a result of negotiation between agents and humans/organizations (Murukannaiah et al., 2020). Cointe et al. (2016) propose a framework that enables an agent to make judgments on whether actions are moral and ethical, not only concerning actions made by the agent itself but also with respect to the actions of other agents. They have identified two requirements needed to design agents that can create such judgments, (i) an explicit representation of ethics, and (ii) an explicit process of creating judgment regarding one’s own actions and those of others agents. These considerations remain relevant in the context of Caesar, however, our framework aims to provide, at a high level, a structured way of factoring in the decision-making ethical aspects. As such, we view our proposal as complementary to what is found in the literature. London and Danks (2018) argue for a regulatory policy for autonomous systems with similar stages as those used for the introduction of medical drugs and other interventions to the general public.

The interaction of a human with a MAS has also raised ethical questions concerning the preservation of human autonomy, given that the process through which a MAS can give information to humans is not always transparent, and can hide viable alternatives which could infringe on the autonomy and liberty for making a decision (Susser, 2019). To address these concerns, Inverardi (2019) have proposed the use of exoskeletons, which guide the development of software designed to act as a protective shell around a human, allowing the human to interact with a MAS on its own terms. The Caesar framework does not prescribe in detail how the interface between the human and agent should be implemented. As such, different approaches are possible, e.g., the exoskeletons proposed by Inverardi (2019) represent a way in which the interface block in Caesar could be specified further, thus guiding the implementation stage.

## Conclusion and Final Remarks

In this paper, we present Caesar, an approach to model ethical aspects for collective decision-making in autonomous systems, with support for accountability. An ethical decision will preserve the desired requirements for security, safety, and privacy. Therefore, these attributes are explicitly modeled in Caesar, and their impact on the collective decision-making is captured not only in the context of one single agent, as proposed in the discussed related works, but more importantly in the context of collective decision-making between different entities.

The main component in the framework is the Caesar agent, which is described in terms of a three-layered structure that addresses, (i) the local decision-making strategies and the role and rank of an agent, (ii) its settings for security, safety, and privacy, and (iii) the computed trust in other agents or humans engaged in the process. As such, Caesar provides, at the level of an individual Caesar agent, a structured approach for reasoning about the information flow between the building blocks that potentially affect the ethicality of a decision. Additionally, the Caesar approach considers a Caesar agent as part of a collective, such as a MAS, where the collective decision-making process shapes the factors that are captured in the building blocks of the agent itself. In this context, the Caesar agent can either be an independent and autonomous actor in the decision-making, a proxy for a human operator, or an entity that is configured at a certain level of autonomy by a stakeholder. The different actors part of such a process will exchange information and interact at different levels, thereby affecting each other’s behavior and individual decision-making processes. Modeling the latter as prescribed in Caesar can help to guide the collective towards an ethical decision. Note that, the end goal of Caesar is not to promote cooperation between agents and/or humans, but instead to promote ethical collective decision-making. To enable the achievement of such a goal, Caesar points out how the software architecture design requires to be adapted to accommodate ethical aspects in collective decision-making.

Finally, we discuss the applicability of the Caesar approach using two illustrative examples from different domains, namely elderly care, and self-driving cars scenarios, where we indicate how such an approach, through the way it models the collective decision-making process, could support tracing back an accountable actor(s). Note that Caesar is not a generic solution to the accountability problem, but rather an enabler. Future work will investigate in-depth the mechanisms with which Caesar can support accountability in a real-world scenario.

## Footnotes

1 In this paper, the terms autonomous system, agent, robot, and vehicle are used interchangeably.

2 The autonomy levels described for self-driving cars (Kyriakidis et al., 2017) are an instance of the 10-levels of automation.

3 The role distinguishes between proxy, peer, subordinate, leader, whereas the rank refers to the titles of the different actors in a given organization, e.g., doctor, nurse, etc.

4 Independently of the ethicality of a decision, in a real-world scenario, the actual outcome resulting from a decision that has been taken, will be affected by the uncertainty of the environment. This aspect is considered outside the scope of this paper, and will be addressed in future work.

5 No assumption is made about the homogeneity of these robots when it comes to vendors, implemented behaviors, autonomy levels, or degree of adaptation.

6 The code is available at https://github.com/struharv/caesar-experiment.
